# Radiographic visualization of magma dynamics in an erupting volcano

**DOI:** 10.1038/ncomms4381

**Published:** 2014-03-10

**Authors:** Hiroyuki K. M. Tanaka, Taro Kusagaya, Hiroshi Shinohara

**Affiliations:** 1 Earthquake Research Institute, The University of Tokyo, 1-1-1 Yayoi, Bunkyo-ku, Tokyo 113-0032, Japan; 2 National Institute of Advanced Industrial Science and Technology, Umezono 1-1-1, Tsukuba, Ibaraki 305-8568, Japan

## Abstract

Radiographic imaging of magma dynamics in a volcanic conduit provides detailed information about ascent and descent of magma, the magma flow rate, the conduit diameter and inflation and deflation of magma due to volatile expansion and release. Here we report the first radiographic observation of the ascent and descent of magma along a conduit utilizing atmospheric (cosmic ray) muons (muography) with dynamic radiographic imaging. Time sequential radiographic images show that the top of the magma column ascends right beneath the crater floor through which the eruption column was observed. In addition to the visualization of this magma inflation, we report a sequence of images that show magma descending. We further propose that the monitoring of temporal variations in the gas volume fraction of magma as well as its position in a conduit can be used to support existing eruption prediction procedures.

## Abstract (web summary)

Visualization of magma in a conduit with classical probes remains challenging due to geological heterogeneity and the geometrical structure of the conduit involved. Tanaka *et al.* use cosmic ray muons and report the first radiographic observation of the ascent and descent of magma along a conduit.

## Graphical abstract

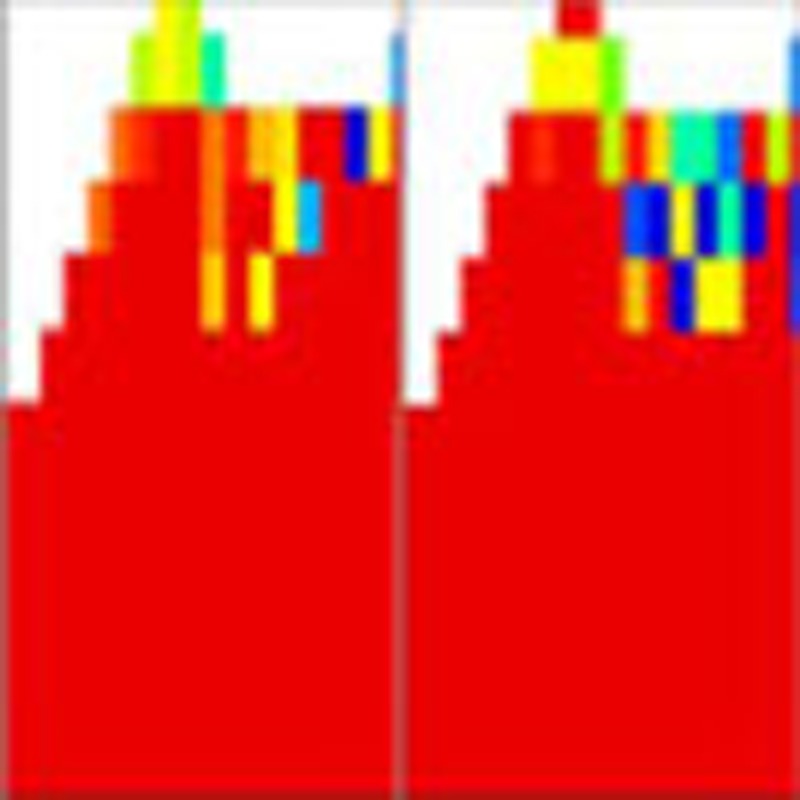

Atmospheric muons are produced via primary cosmic rays reacting with the Earth’s atmosphere and their energy spectrum is well known. Since the atmospheric muon’s penetration flux (the muon flux after passing through a given thickness of rock) is simply influenced by the muon path length and the density along the path, if we place a muon detector at the foot of a mountain, we can predict that there will be deviations from a typical muon spectrum free from geological obstacles (open-sky spectrum). This deviation can be precisely calculated, and the theoretical uncertainty in these calculations is small^1^. Thickness as well as density of the target object will therefore determine the number of muons that will successfully pass through the rock and reach the detector.

In general, visualization of magma in a conduit with classical probes (for example, earthquakes and electric current) remains challenging owing to intrinsic complexity, such as the geometrical structure of the conduit involved and geological heterogeneity. Unlike classical probes, the advantage of the muon’s ray path is that it is straight and thus accurately identifiable. We can therefore improve the spatial resolution of an image simply by gaining the tracking resolution of the detector. However, the technique is limited to horizontal ranges of 2–3 km (which limits the potential targets). This technique only resolves the average density distribution along individual muon paths. Therefore, the user must end up making assumptions or interpretations about the internal structure, or must use more than one detector to resolve the three-dimensional (3D) structure.

One of the most historically well-known examples of muography measurements is the experiment performed by Alvarez *et al.*^2^ The goal of their experiment was to attempt to discover secret chambers inside the Khafre’s pyramid with muography. The internal structure of the peak region of the Asama volcano was first imaged with muography in 2006 (ref. 3), and the technique was found to have the potential to spatially resolve the internal structure of a volcano with a higher resolution than possible with the conventional geophysical techniques, as well as to support confirmation of data from other techniques (for instance, the shape of the magma deposits in the crater estimated with airborne synthetic aperture radar (SAR) measurements both before and after the 2004 eruption)^3^. Additionally, their measurement imaged a low-density region underneath the crater floor, which was interpreted as a magma pathway plugged by magma deposits on the crater floor. Subsequently, Tanaka *et al.*^4^ collected additional muography data from Asama that supported the conclusions of the previous survey in 2006. The muography monitoring system captured images both before and after the 2009 Asama eruption, and the disappearance of material in the northern section of the magma deposit was detected. A petrological study of the 2009 eruption ejecta showed that it matched the old magma deposit created in the 2004 eruption, and provided verification of this disappearance.

To investigate the conduit diameter and shape, a muography measurement was targeted underneath the Showa-shinzan lava domes of Usu volcano^5^,^6^. The diameter of the magma pathway was consistent with the Yokoyama model^7^,^8^,^9^. Visualization of a conduit has also been attempted in the Puy de Dome volcano, France^10^, which imaged a similar bulbous magma structure underneath the surface. Inside the lava dome of La Soufrière, France, a strongly inhomogeneous density distribution was muographically observed^11^. A high-density rock layer was indicated in between the hydrothermal region and the deeper region. If this region is overpressured when the geothermal energy flux increases, it may explode in an episode similar to what Tanaka *et al.*^3^ observed in the Asama eruption.

Magma in a conduit is a dynamic entity, and its motion is essential to the eruption process. Visualizing dynamic magma processes is often a key component to apply towards understanding the eruption patterns^12^,^13^,^14^,^15^. Thus, time-sequentially captured radiographic images (muographs) provide a wealth of detailed information that can yield significant insight into eruption dynamics. Muography is a practical method for testing a volcanological hypothesis; for instance, magma significantly inflates near the vent where the pressures are low and the associated fragments are accelerated to high speed before an eruption; the inflation degree will determine whether an explosive eruption will occur^15^.

The Satsuma–Iwojima volcano continuously discharges large amounts of volcanic gases without significant magma discharge. One of the proposed mechanisms of this continuous gas discharge is conduit magma convection^16^. Since an oversaturation of volatiles in a melt sample was found, it was expected that the degassing only occurs in relatively low-pressure conditions^17^. In 2008, the muography technique imaged a large (390–450 m in diameter), shallow depth (150–200 m from the crater floor), low-density (<1.0 g cm^−3^) region existing beneath the crater floor^18^,^19^. Degassing magma, with its high proportion of bubbles, has been interpreted as being the low-density region in the resulting muograph. The depth of the magma head observed 150–200 m below the crater floor is consistent with the magma degassing pressure of 0.5–3.0 MPa in Satsuma–Iwojima, as estimated by laboratory and theoretical studies^20^. This muographic image suggests that the low-density region at the top of the magma column is a common feature of conduit magma convection.

It is generally expected that as water-rich magma ascends to the ground surface and decompresses, volatiles are exolved and magma becomes less dense. We developed a muon detector with the lowest noise levels to visualize and analyse magma movements and modification patterns in order to directly test this hypothesis. In this paper, we report the magma dynamics during two eruptive episodes in Satsuma–Iwojima volcano, Japan, that were captured by a muon detector operated at the lowest noise level.

## Results

### Observation

**Figure 1:**
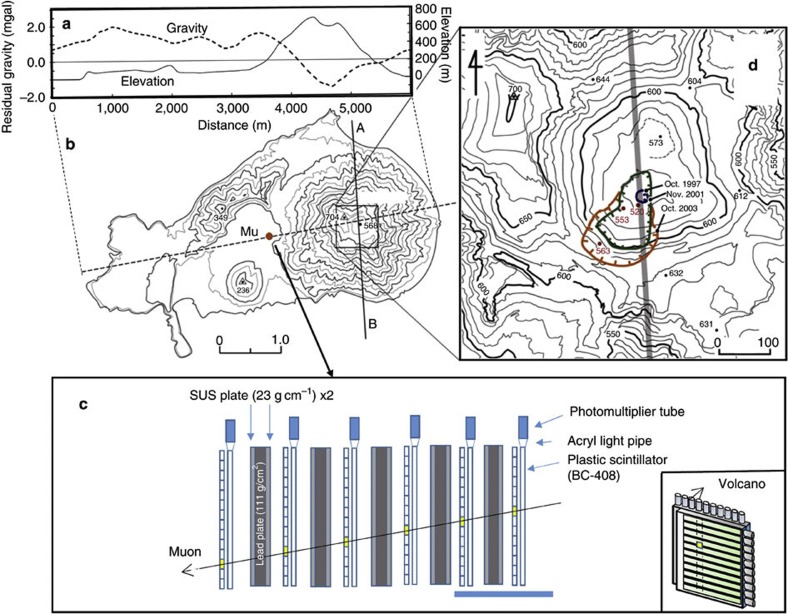
Experimental set-up of current muographic observation system. (**a**) A profile of residual gravity and topography sliced along the dotted line in (**b**) is derived from Bouguer anomalies of an assumed density of 2.0 g cm^−3^ and trend removal of upward-continuation of 500 m (ref. 19) based on the gravity data set obtained for the published map. (**b**) The topographic map of Satsuma–Iwojima volcano shows the location of the muon detector (indicated by Mu). A topographic profile along the A–B line was created to support muographic images.(**c**) A schematic view of the detector was used for the present observation. It consists of five lead plates supported by stainless steel plates and six layers of scintillation PSPs. Scale bar 1 m. The inset shows the layout of the PSP consists of adjacent scintillator strips, which together form a segmented plane. (**d**) Close-up map of the summit area of the cone in the box on the map labelled as (**b**). This enlarged map shows newly created inner crater between 1997 and 2003 (blue, green and orange lines). The grey line on the map corresponds to the A–B line shown in (**b**).

We developed a muon detector with the lowest noise levels to visualize and analyse magma movements and modification patterns in Satsuma–Iwojima volcano, Japan. Satsuma–Iwojima volcano (Fig. 1a), composed of cooled thick lava flows and corresponding collapsed deposits, discharges a large amount of volcanic gases with SO_2_ flux of 500–1,000 t day^−1^ (ref. 21). SO_2_ flux and volcanic gas composition have been almost constant for at least the last 30 years, and this rate has likely been constant for >100 years (ref. 21). A new vent formed in 1997 and by 2003 had enlarged to become an internal crater (Fig. 1d). On 4 June 2013, the eruption alert level had risen from level 1 (signs of volcano unrest) to level 2 (minor eruptive activity). On 13 June, it was detected that the vent size increased. The eruption sequence of 2013 Satsuma–Iwojima eruption is shown in Table 1. The muon detector was placed ~1.4 km from the summit crater (Mu in Fig. 1a) on 14 June 2013.

**Figure 2:**
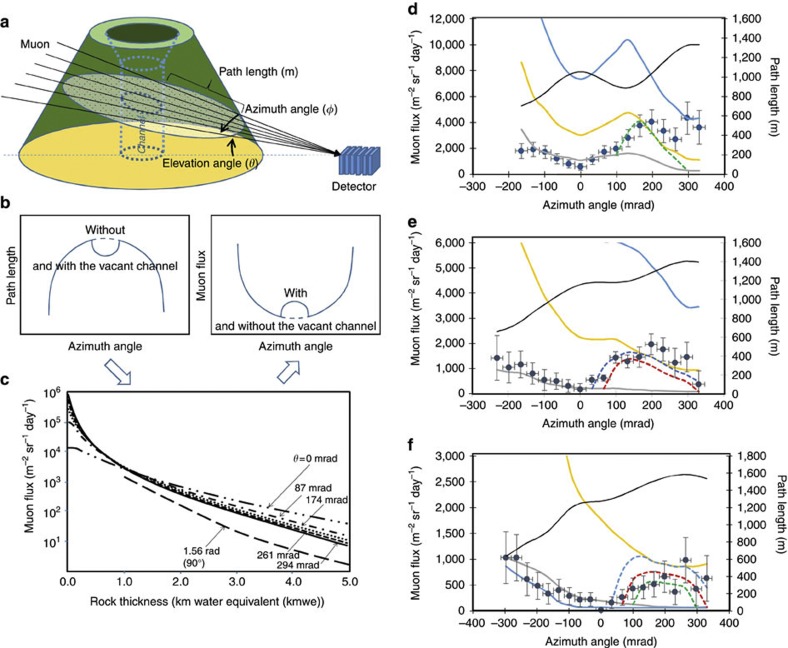
Principle of muographic measurements. (**a**) Geometrical arrangement of the detector facing a volcano defines azimuth and elevation angles, as well as path lengths. The path length distribution as a function of azimuth angle is converted to the penetrating muon flux distribution (**b**) via the flux–thickness relationship (**c**). (**c**) The plot shows the flux of muons that have enough energy to continue traversing through a given thickness of rock in units of kmwe (penetrating muon flux), and compares the penetrating muon flux arriving at various elevation angles in units of radian. The data are from Fig. 1 of ref. 6, apart from the 1.56-ad curve. The measured muon flux in Satsuma–Iwojima from the direction of elevation angles of (**d**) 297±16.5, (**e**) 264±16.5 and (**f**) 231±16.5 mrad is included. The measured flux is compared with theoretical flux calculated for various given densities (0.7–2.5 g cm^−3^). Path length distribution is also plotted for reference. Error bars represent 1 s.d. of statistical uncertainty (1 s.e.). The s.e. was calculated for muon counts in each bin.

Muons are collected as a function of azimuth (*φ*) and elevation (*θ*) angles after they have passed through the mountain (Fig. 2a). Concurrent to the data from the detector, topographic map-derived path length information is used for reconstructing muon flux for various angles (*φ*, *θ*) and for a given uniform density (*ρ*) by using a relationship between the penetration muon flux and the rock thickness through which the muon traverses (Fig. 2b,c). Size and density of the target volume will determine the number of muons that will successfully pass through the target volume and reach the detector. The flux of atmospheric muons after passing through a given thickness of rock (penetrating muon flux) can be theoretically predicted as a function of the arriving angle (Fig. 2c). Comparisons between the actual muon flux and the expected muon flux for various densities of the body yield density length distributions as a function of *φ* and *θ*.

The muon absorption rate therefore directly measures the density length (density × muon path length). Once the path lengths are calculated from topographic information, average density <*ρ*> can be determined along the path lines of the cosmic-ray muons. As the density length increases, the penetrating muon flux decreases, and we find that muons penetrating targets thicker than 2 km water equivalent (kmwe) (equivalent to 1-km rock if <*ρ*>=2 g cm^−3^) are infrequent events. In order to collect these rare events, we need a detector with a large active area, typically >1 m^2^. For example, from the total number of horizontal muons passing through rock with a thickness of 2 and 4 kmwe respectively, we could expect that a muon detector records 10^3^ and 10^2^ muons m^−2^ sr^−1^ day^−1^. There are different versions of large-scale muon detectors including plastic scintillator-based^22^, liquid scintillator-based^23^, gaseous^24^, water Cerenkov^25^ and emulsion-based^3^ detectors.

During muography measurements, the most prominent class of backgrounds comes from electromagnetic (EM) components. In situations where the muography system consists of two position-sensitive planes, the major source of the fake muon tracks is the accidental coincidence of vertical EM shower particles^26^. In order to effectively discard such accidental events, redundant counters are often added to the system. The background noise or the fake tracks initiated from horizontal high energy electrons and low energy muons can be reduced by inserting a thick steel or lead shield in between the planes. The configuration for the Satsuma–Iwojima detector has four redundant planes.

The muon detector consists of five 10-cm (111 g cm^−2^) thick lead plates supported by ten 3-cm (23 g cm^−2^) thick stainless steel plates and six layers of scintillation position-sensitive planes (PSPs) (Fig. 1e). Its dimensions are 3 × 1.7 × 1.7 m^3^, with the lead and steel plate thickness totalling 50 cm (~555 g cm^−2^) and 30 cm (~230 g cm^−2^), respectively. Each position-sensitive plane consists of *N*_x_=14 and *N*_y_=14 adjacent scintillator strips, which together form a segmented plane with 14 × 14 segments. The total area of the scintillator part corresponds to the active area, and the distance between the first and sixth planes is 3 m. The detector is triggered by a signal generated by a single muon passing through the following layout: six layers of PSPs having a 1.4 × 1.4 m^2^ active area with a spatial resolution of 10 cm, that is, and an angular resolution of 33 mrad (~1.9°). This angular resolution is equivalent to the spatial resolution of 46 m at the centre of the cone. When a muon passes through the detector, a vertex is created on each PSP component of the sixfold cosmic ray muon detector. All vertices are considered but only the vertices that are aligned along a straight line are exploited in order to ensure that only muons that passed through the volcano are selected. Adding the lead shield increases the apparent density length (8 mwe) of the target volume. However, this increase is negligible in comparison to the typical density length of our target, which ranges from 1,000–2,000 mwe.

This system was designed for rejecting EM components as well as low-energy muons (less than a few GeV) that can be deflected in the space between the mountain and the detector, and thus could deliver the wrong directional information. Root mean square (r.m.s.) scattering expected for muons with energies of 0.3 and 3 GeV passing through 50 cm of lead are 472 and 47.2 mrad respectively. In order to prevent such low-energy muons from triggering the detector, the event is considered valid only when the six intersection points make a linear trajectory. This enhanced background reduction dependent on the thick lead and iron plates and large number of redundant layers for muon tracking enabled us procure an extremely low background rate <10 sr^−1^ m^−2^ day^−1^, and as a result, time resolution for imaging was drastically improved. The angular resolution and active area are 33 mrad and 2 m^2^, respectively. It is worth commenting here on the background noise, which results in degrading the time resolution of the observation. If the number of background events *N*_BG_ is larger than true muon events (*N*), the statistical error of background events (*N*_BG_)^1/2^ will seriously mask the muographic image, and therefore a larger number of events will be necessary to record until the modulation in the penetrating muon flux (Δ*N*) due to the density contrast (Δ*ρ*) in the target volume becomes larger than (*N*_BG_)^1/2^. Thus, the reduction of background events is essential for rapid time sequence muography. Spatial resolution of the target volume is determined exclusively by the angular resolution (30 mrad) of the detector and the distance between the detector and the mountain. Therefore, the spatial resolution will be the same at the top of the conduit as it is at a 1-km depth. However, the density resolution is degraded as the thickness of the target increases, since the muon counting rate is reduced (Fig. 2c): lower counts yield higher statistical fluctuations.

The size of the detector is negligible relative to the length scales of a volcano; therefore, we use a coordinate system in which each point on a target volume is determined by an angle and distance. The positioning resolution at the target volume is determined by the angular resolution of the detector (Δ*θ*) and the distance to the target (*L*): *L* Δ*θ*. For instance, the positioning resolution at the crater of the cone is 46 m, since the distance to the crater is 1.4 km. The distribution of the density length ((density length) × (average density along the muon path)) is theoretically calculated from a penetrating muon flux as a function of the rock thickness through which muons traverse^6^. The average density along the muon path is then mapped within an angular coordinate as a function of elevation (*θ*) and horizontal angles (*φ*) (Fig. 2a). When an excess count of transmitted muon events is observed, the density of the target is seemingly lower than the presumptive density. It is then assumed that such a low-density region is localized around the vent area and the diameter of the vent is calculated based on the muographically determined density distribution. By comparing the average density of the surrounding rock (which is also determined with the same procedure) and that of the vent area, the average density inside the vent can be deduced.

### Analytical results

Figures 2a,e,f show the correlation between the azimuth distribution of the measured penetrating muon flux for the entire observation period and the expected muon flux for various densities. Additionally, the path length as measured from the topographic map is also plotted. By comparing the measured and theoretical muon flux side by side, a systematic deviation can be clearly seen in all of the plots (−132 mrad<*φ*<330 mrad, −66<*φ*<330 mrad and −66<*φ*<330 mrad for *θ*=297±17 mrad, 264±17 mrad and 231±17 mrad, respectively). Shinohara and Tanaka^19^ reported that a low-density conduit with a diameter that ranges from 390–450 m was detected located underneath the crater floor of Satsuma–Iwojima independently with a different type of the muon detector. Therefore, this deviation is assumed to be the low-density conduit, and the average density of the mountain body is calculated by avoiding this region. The values of these densities are 1.68 1.86 and 2.2 gcm^−3^ for *θ*=297±17 mrad, 264±17 mrad and 231±17 mrad, respectively. Subsurface density structure is commonly estimated with a gravimetric survey. As shown in Fig. 1c, gravity mapping of Satsuma–Iwojima (with a residual profile derived from Bouguer anomalies of an assumed density of 2.0 g cm^−3^) revealed that a low gravity region was located within the volcanic cone, which reached its lowest value at the crater region^27^. As the muograph measured the density of the cone to be ~2.0 g cm^−3^, negative residual gravity values implied the existence of a very-low-density body in the cone, and the spatial extent of the low gravity region was consistent with the muographic measurement of the conduit size.

Although techniques of seismic tomography have been applied to imaging the internal structure of volcanoes, most of the studies have focused on the velocity structure of the entire volcano, mapping the possible location of melt material or a shallow magma reservoir^28^,^29^. These tomographic measurements have a horizontal extent of 10–20 km, a depth of investigation of a few kilometres and a spatial resolution of the order of 1 km. A decametric resolution to resolve much finer details requires extremely high recording densities. Recently, Brenguier *et al.*^30^ conducted seismic tomography of a conical-shaped volcano with a base diameter of 1 km (approximately the size of Satsuma–Iwojima) and obtained a spatial resolution of 80 m by creating 400 seismic sources (370 sites for 16-ton vibrators and 30 sites for dynamite shots), having received the signals with a High Resolution Imaging Array^31^ consisting of 258 seismic sensors. A tomographic measurement of this kind would be both economically and practically out of reach in the harsh field conditions in Satsuma–Iwojima. However, other kinds of seismological studies have provided useful information on the internal structure of Satsuma–Iwojima.

Ohminato^32^ and Iguchi *et al.*^33^ showed that low-frequency earthquakes can be a useful tool to explore the source mechanism of volcanic tremors in Satsuma–Iwojima. Ohminato showed that very-long-period (VLP) seismic pulses synchronizing with modulation in the volcanic tremor amplitude (which are thought to be related to volcanic conduit magma convection in Satsuma–Iwojima) can be explained by the rapid expansion of a gas pocket located 100 m underneath the crater floor^32^. A similar model was suggested by Iguchi *et al.*^33^ to explain the 1996 earthquakes and fault creation in Satsuma–Iwojima. The low-density region detected underneath the crater floor with muography (150–200 m beneath the crater floor) is consistent with both the numerical^34^ and these seismological estimates.

**Figure 3:**
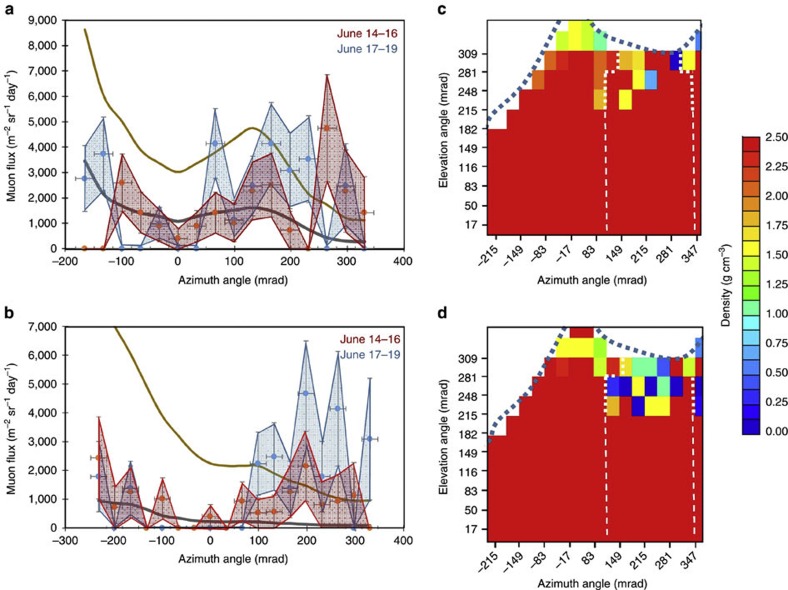
Comparison of muographic measurement results. The plots compare the muon flux measured in different time periods: 14–16 June (red dots) and 17–19 June (blue dots) for different elevation angles (297±16.5 mrad (**a**) and 264±16.5 mrad (**b**)). Theoretical flux calculated for various given densities (1.0 and 2.0 g cm^−3^) is also shown for reference. The shaded area indicates 1*σ* (68% CL) zone. Error bars represent 1 s.d. of statistical uncertainty (1 s.e.). The s.e. was calculated for muon counts in each bin. 2D presentations of the 1*σ* (68% CL) upper limit of the average density along the muon paths (**c** and **d**) are compared between the observation period of 14–16 June (**c**) and 17–19 June (**d**). The blue bold dashed line indicates the outline of the cone along the A–B line in Fig. 1b. The conduit diameter determined at an elevation angle of 264±16.5 mrad was extrapolated to an elevation angle of 0 mrad (white broken line). The elevation and horizontal distances at the centre of the cone are also shown in (**c**) and (**d**) along with the elevation and azimuth angles.

In the case of two-dimensional (2D) (uni-directional) radiographic projection, the most straightforward visualization is to assume a cylindrical conduit. While often useful, this method has obvious limits. However, it is still helpful for researchers to use in order to calculate magma flow rate in a conduit as well as temporal changes in overall gas volume fraction. By assuming the existence of a cylindrical conduit, the best-fitting gives diameters and densities of (*d*,*ρ*)=(280 m, 0.0 g cm^−3^) (*χ*^2^/*n*=1.05), (*d*,*ρ*)=(470 m, 0.0 g cm^−3^) (*χ*^2^/*n*=0.99) and (*d*,*ρ*)=(460 m, 0.9 g cm^−3^) (*χ*^2^/*n*=1.71) for *θ*=297±17 mrad, 264±17 mrad and 231±17 mrad, respectively. The density and diameter values are consistent with the values reported by Shinohara and Tanaka^19^ for Satsuma–Iwojima: ((*d*,*ρ*)=(170 m, 0.4 g cm^−3^) for 300±20 mrad and (*d*,*ρ*)=(390–450 m, 0.9 g cm^−3^) for 240±40 mrad) and are also consistent with the Nicaraguan Masaya volcano, which has a conduit diameter of 440 m, a similar volcano to Satsuma–Iwojima in that it is a persistently degassing volcano^35^,^36^. Therefore, we will use these depth-dependent conduit diameters for the following analysis (indicated by dashed white lines in Figs 3 and 5). Two data points for *θ*=297±16.5 mrad within the azimuth region between 267 and 330 mrad are found to be strongly deviated from the density curve for 1.7 g cm^−3^. The region corresponds to the location of a newly created (between 1997 and 2003) crater (Fig. 2d)^37^, which was not considered when calculating the muon path length in the present discussion.

In general, it is difficult to perform rapid time sequence radiography because the relatively low intensity of the cosmic ray muon flux requires long integration times to obtain adequate muon transmission image contrast. However, to survey the large-amplitude and low-frequency motion of magma in a small-scale volcano such as Satsuma–Iwojima, radiographic methods measuring time-dependent changes in the muon flux after passing through the conduit region can provide an efficient approach. Our detector is capable of collecting 1.75 muons per day in each bin (33 × 33 mrad^2^) after passing through 800-m rocks (*ρ*=2.0 g cm^−3^) from the direction of 264±17 mrad. If the volcano conduit is filled with magma of the same density, this path length will increase to 1,200 m and the muon count will be reduced to 0.37 muons per day. This difference between an empty or full conduit can be detected in 3 days at a 2*σ* (95%) confidence level (CL).

**Figure 4:**
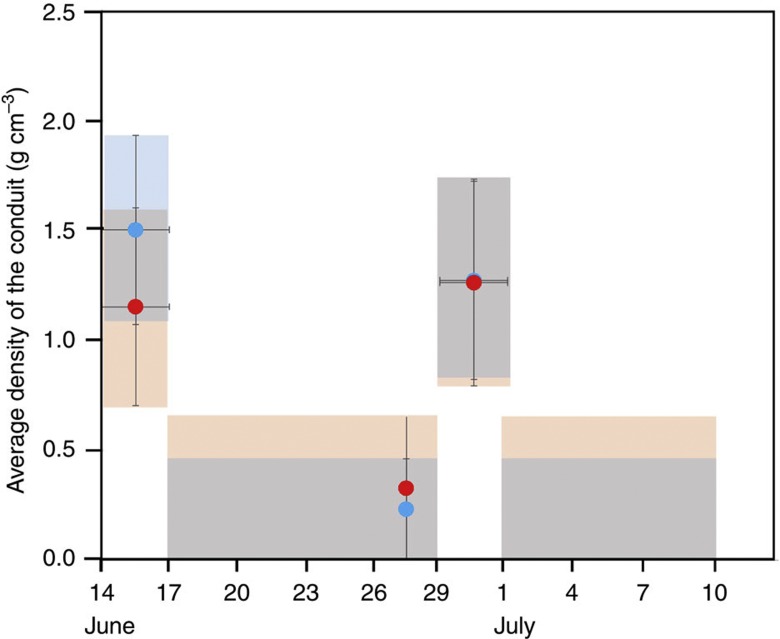
Time-dependent average density of the conduit. The values as measured during the periods of 14–16 June, 29 June–1 July, are compared with the entire period excluding these two periods for different elevation angles: 297±16.5 mrad (blue dots) and 264±16.5 mrad (red dots). Error bars represent 1 s.d. of statistical uncertainty (1 s.e.). The s.e. was calculated for total muon counts recorded during each period. The blue and orange-shaded areas indicate 1*σ* (68% CL) zone for each elevation angle.

Figure 3a compares the azimuth distribution of the muon flux from the 14–16 June measurement with the 17–19 June measurement, both from an elevation angle region of 297±16.5 mrad. On 16 June, a white column of volcanic steam and ash was observed rising up to 400 m above the crater rim (Table 1). The shaded areas correspond to the 1*σ* (68% CL) zone of the data points. Figure 3b shows how the same plot varies for different elevation angle regions (264±16.5 mrad). From these plots, the 1*σ* upper limit of the average density along the muon path can be calculated for each bin (Fig. 3c,d). The figures are superimposed on the outline of Iwojima volcano, which is cut by a plane along the A–B line in Fig. 1a (dotted line). Two vertical dotted lines indicate the region of the conduit. The colour mapping inside this region indicates the upper limit of the average density along the muon path and within the conduit by assuming a surrounding rock density of 1.68, 1.86 and 2.2 g cm^−3^ for *θ*=297±17, 264±17 and 231±17 mrad. Significant differences exist between the 3-day period of data collection during and after the volcanic eruption (during which the white column was observed) as can be seen in Fig. 3c,d. Figure 3c,d shows a muographic snapshot sequence of Satsuma–Iwojima volcano. The frame rate is 10 frames per month (FPM). Figure 3c,d plot the 68% confidence level (1*σ* CL) of the upper limit of the density. This means that there will be still a statistical fluctuation in these presentations. In order to evaluate these variations at higher confidence levels, the bins are packed and compared in Fig. 4. Figure 4 shows the time-dependent changes in the flux of the muons after passing through the conduit region for two elevation angles (*θ*=297±17 and 264±17). In order to achieve higher and more accurate statistics, the bins are packed within the azimuth region of the conduit (116 mrad<*φ*<248 mrad) and thus the density values are averaged over this azimuth region in this plot. The surrounding rock density is determined as described above. The shaded area indicates the 1*σ* zone. Average density measured during 14–16 June and 29 June–1 July deviates by >2*σ* (95% CL) from the average value over the entire period excluding above periods (17–29 June and 1 July–10 July).

## Discussion

Our interpretation of Figs 3c,d and 4 is that before 14 June, the top of a convecting magma column ascended to a level that was 60 m beneath the crater floor (10 m beneath the newly created crater floor (Fig. 1d)), and that during the period from 14–16 June, the condition remained the same. On 16 June, a white-colored eruption column was ejected from the vent, rising to a height of 400 m above the crater rim. During night-time of the same day, a volcanic glow was observed. After the eruption, the top of the magma column descended (17–19 June). Matsushima^34^ reported that a shallow degassing at a depth lower than 300 m is necessary to create the geochemical observation results. Therefore, we assume that the low-density (*ρ*=1.0–1.5 g cm^−3^) material filling the conduit during 14–16 June and 29 June–1 July was the shallow top of the convecting magma column. The magma convection rate of Iwojima was estimated to be 1 × 10^6^ m^3^ day^−1^ on the basis of the SO_2_ emission rate of 550 t day^−1^ (ref. 20). If the inner ascending radius is 200 m, the average flow speed will be 10 m day^−1^ for low-density, vesiculated magma (assuming 1 g cm^−3^). On the other hand, Fig. 4 indicates that the average flow speed is 30 m day^−1^ (100 m in 3 days).

**Figure 5:**
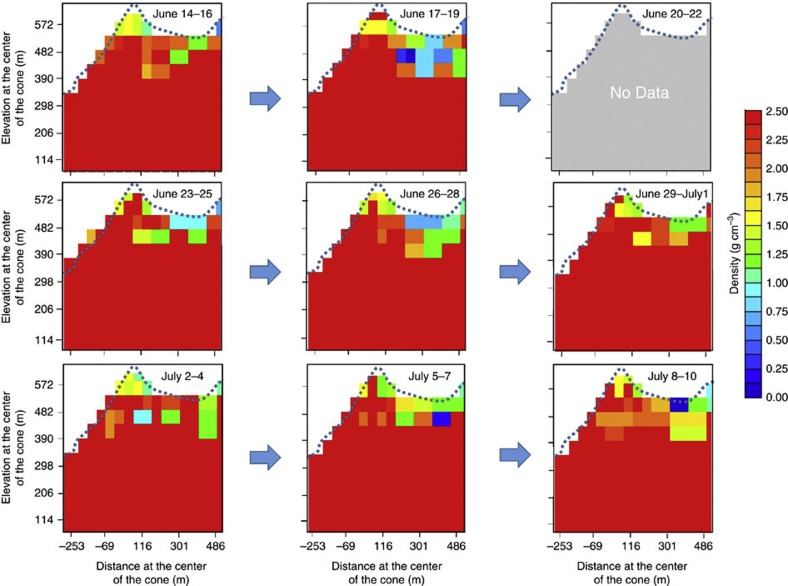
Time sequential muographic animation. The plots show the angular distribution of 1*σ* (68% CL) upper limit of the average density along the muon path. The frame rate is 10 FPM. The data were not taken during 20–22 June due to a blackout. Horizontally adjacent two bins were packed in order to achieve higher and more accurate statistics. The elevation and horizontal distances at the centre of the cone are shown.

Figure 5 shows a muographic animation with a frame rate of 10 FPM demonstrating the magma dynamics until the eruption warning was cancelled by JMA on 10 July by plotting a 1*σ* CL (68% CL) upper limit distribution of the average density along the muon path. The same procedure to produce Fig. 3c,d was applied for the calculation of the density distribution inside the conduit. Supplementary Movie 1, based on the data in Fig. 5, exhibits how the different density regions change with time, and is also synchronized to the visual observations of the eruptive phenomena (graphically retouched based on the data shown in Table 1). Since a lightning strike led to blackout in the area, the data were not collected from the 19th to the 22nd of June (Fig. 5c). During the period from 29 June to July 1st, we also observed similar phenomena: magma ascended in a conduit and the highest part of the magma column reached the same height as 14–16 June. Once more, on 30 July, a white-colored eruption column was ejected again and reached 200 m above the crater rim. Volcanic glow was also recorded throughout the night of 30 July. As shown in Fig. 5, the magma column does not ascend so fast, while the gaseous region above it seems to be more dynamic. Based on these data, when the eruption column is created, the shape of the low-density region appears to change while the denser region below remains at about the same level in the conduit.

This study provides the first muographic visualization of magma column dynamics in an erupting volcano. The reported muographic observation results confirm the movement of the top of a magma column before and after two eruptive episodes. While the eruption column was observed (although it does not have to correlate with increased degassing), the top of the magma column reached a location of 60 m beneath the crater floor. This shallow magma body heated the crater floor and exhibited a volcanic glow event during the night-time of the same day. The ascending speed of the low-density, vesiculated magma body at the top of the magma column seems faster than that of the higher density magma body. During the process of developing the low-noise detector, we successfully removed several empirical factors (for example, manual background reduction from data) from the analysis procedure. However, the complex user interfaces in the apparatus still present a challenge to many scientists who would otherwise benefit from integrating muography data into their research. In the near future, we anticipate more effective computational approaches for analysing and presenting data. Increased collaboration with the volcanology community will also lead to the development of more practical and intelligible approaches.

## Methods

### Apparatus

Our assembly-type muography detector consists of 168 counter bars, 1 muon readout module, 1 power supply (Matsusada Precision HAR-2N300) and 10 noise absorber modules. The counter bar consists of a plastic scintillator (Bicron BC-408) bar connected to a photomultiplier tube (PMT; Hamamatsu H7724) via an acryl light pipe with the components measuring ~2 m in length and weighing 3 kg in total^22^. Weighing ~5 kg with ~30 W power consumption, the muon readout module is situated on the side of the detector unit^38^. The noise absorber module weighs 1.5 tonnes, making it the heaviest module. All modules can be transported separately and assembled at the observation site. Assembling time for this detector configuration was accomplished in 24 h with the assistance of five people.

## Author contributions

H.K.M.T. wrote the main manuscript text and prepared Figs 3, 4, 5. T.K. prepared Fig. 2. H.S. prepared Fig. 1. All authors reviewed the manuscript.

## Additional information

**How to cite this article:** Tanaka, H. K. M. *et al.* Radiographic visualization of magma dynamics in an erupting volcano. *Nat. Commun.* 5:3381 doi: 10.1038/ncomms4381 (2014).

## Supplementary Material

Supplementary Movie 1 Muographic animation of Satsuma-Iwojima. The time-sequential muographic images are synchronized to the visual observations of the eruptive phenomena. The frame rate is 10 frames per month (FPM).

## Figures and Tables

**Table 1:** Sequence of the 2013 Satsuma–Iwojima eruption.

**Date June**	**4**	**6**	**7**	**16**	**17**	**30**	**July 10**
Column height (m)	—	300	600	400	100	200	—
Volcanic glow	—	—	—	X	—	X	—

**Remarks**	**Lv2 eruption**	**Lv2 eruption**
	Warning issued	Warning cancelled

The data were taken from The JMA (Japan Meteorological Agency) Report about Volcanism—Satsuma–Iwojima Vol. 7 (ref. 39). X marks in the table show that the volcanic glow was observed during the nighttime on the corresponding day. The column heights are eruption plume heights.
